# Comparison of endoscopic and microscopic tympanoplasty in patients with chronic otitis media

**DOI:** 10.1007/s00405-022-07273-2

**Published:** 2022-02-05

**Authors:** Qimei Yang, Bing Wang, Jin Zhang, Hui Liu, Min Xu, Wen Zhang

**Affiliations:** Department of Otorhinolaryngology-Head and Neck Surgery, Shaanxi Provincial People’s Hospital, Xi’an, 710068 China

**Keywords:** Endoscope ear surgery, Microscopic, Tympanoplasty, Chronic otitis media

## Abstract

**Background:**

To compare the results between type I tympanoplasty performed with transcanal endoscopic ear surgery (TEES) and microscopic ear surgery (MES) for treatment of chronic otitis media in a homogenous group of patients.

**Methods:**

A retrospective study was performed in our department between January 2011 and January 2016 to review primary type I tympanoplasty cases with a subtotal perforation, an intact ossicular chain, a dry ear for at least 1 month, normal middle ear mucosa, and a follow-up period of at least 6 months post surgery. The adoption of TEES or MES was divided temporally (before and since 2013). TEES was undertaken in 224 patients (224 ears) and MES in 121 patients (121 ears).

**Results:**

The successful graft take rate was 94.64% (212/224) in the TEES group and 90.91% (110/121) in the MES group (*P* = 0.239). The improvements in the air conduction levels between the 2 groups were not statistically different at 1, 3, or 6 (> 6) months (*P* > 0.05) after surgery. The improvements in the air–bone gaps were not significantly different between the 2 groups (*P* > 0.05). The average hearing gains in the TEES group 6 (and > 6) months post surgery were 11.85 ± 5.47 dB, which was better than 10.48 ± 5.18 dB in the MES group (*P* = 0.031). The use of medical resources was lower in the TEES group than in the MES group regarding the average operating time (49.22 ± 8.24 min vs 81.22 ± 14.73 min, respectively; *P* < 0.0001). Patients receiving MES had a significant higher incidence of the wound problems (ear pain, numbness around the ears, and wet ear; *P* < 0.05).

**Conclusion:**

TEES for type I tympanoplasty seems to achieve a shorter operative time and ideal tympanic membrane healing rate and hearing results in patients with chronic otitis media.

## Introduction

Type I tympanoplasty, or myringoplasty, is an effective measure to repair the tympanic membrane and enable the recovery of hearing loss in case of chronic otitis media [1]. It is typically performed via microscopic ear surgery (MES) worldwide [2]. The microscope equipment offers binocular vision along with an excellent stereoscopic surgical view and leaves both the surgeon’s hands free, but it is limited by the straight-line vision that makes visualization of the middle ear through the ear canal relatively difficult. The microscopic surgery requires an upside-down conical “working space,” resulting in much more soft tissue resection, with or without drilling of the bone to obtain adequate illumination [3].

Since the late 1990s, some physicians have successfully used endoscopes instead of microscopes for partial ear surgery [4]. The endoscope for middle ear surgery has been a highly contentious subject with the introduction of high-quality narrow-diameter endoscopes, ultra-high-definition cameras and screens, and cold light sources [5]. In contrast to the microscope, the endoscope has a cone-shaped source of illumination, which ensures optimal visualization and allows a wider field of view of the surgical area [6]. Endoscopic ear surgery permits a wide-angle view throughout the external ear canal, including magnification of the middle ear structures and direct visualization of hidden areas [7], such as the sinus tympani, hypotympanum, posterior part of the mesotympanum, and epitympanum [8].

In this retrospective study, we compared the results of type I tympanoplasty performed with transcanal endoscopic ear surgery (TEES) versus MES for treatment of chronic otitis media with a subtotal perforation, an intact ossicular chain, a dry ear for at least 1 month, and normal middle ear mucosa, over a postsurgical follow-up period of at least 6 months. We then evaluated whether the endoscopic approach offered advantages over conventional surgery.

## Materials and methods

### Study designs

This retrospective study was performed to assess functional results in patients with chronic otitis media who had undergone type I tympanoplasty at Shaanxi Provincial People’s Hospital between January 2011 and January 2016. This study was approved by the Institutional Review Board of the Shaanxi Provincial People’s Hospital.

A total of 682 patients underwent primary tympanoplasty during this period, with all surgeries having been performed by experienced otologist (W. Zhang). The study included a homogeneous group of patients with a subtotal perforation, a dry ear for at least 1 month, an intact ossicular chain, and normal middle-ear mucosa.

We excluded from the study patients younger than 18 years of age; those requiring ossiculoplasty, atelectasis or mastoidectomy; those with obvious narrowing of the external ear canal or syndromes that can affect the middle ear (e.g., Down syndrome); and those with a history of previous otological surgery.

A total of 345 patients were enrolled in the study. Patients were divided into 2 groups based on whether they received TEES (224 ears, 224 patients) or conventional MES (121 ears, 121 patients). The use of MES or TEES had a clear temporal division (before 2013 versus 2013 and after). As of January 2013, TEES was the primary procedure, and MES was used as a salvage technique in cases in which TEES was not suitable, such as in patients with ear canal stenosis.

All surgical procedures were performed by the senior author (W. Zhang). Postoperative follow-up evaluations and management were performed by the intermediate authors. The review of medical records and the data collection and analyses were performed by the first author and research assistants.

We recorded information on the patients’ operative time, successful tympanic membrane healing, postoperative complications, and their pre- and postoperative air conduction pure tone average (PTA), bone conduction PTA, and air–bone gap (ABG).

### Surgical procedures

**Fig. 1:**
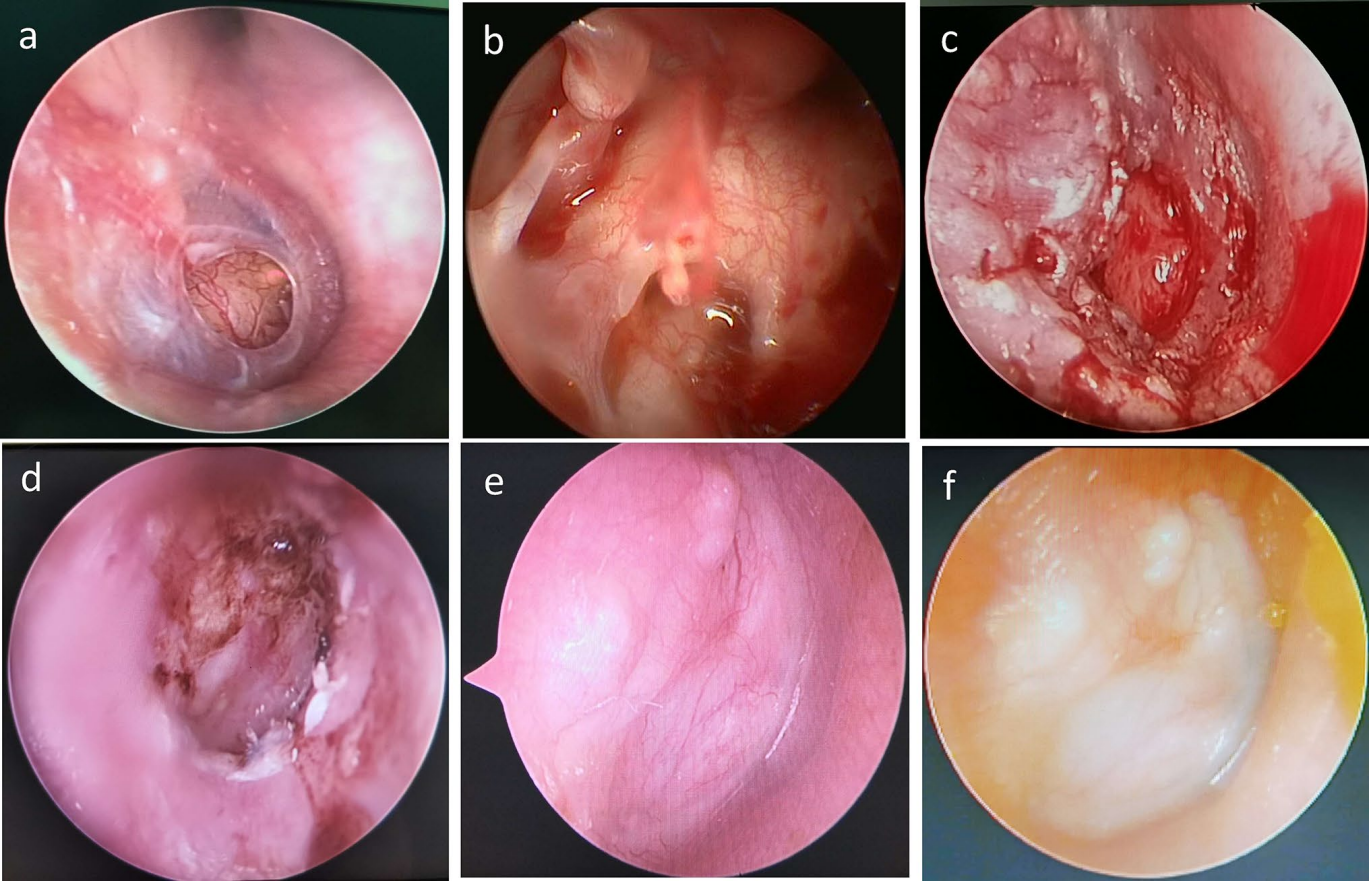
Surgical steps and outcomes of TEES images (right era, **a**–**f**). Subtotal perforation of the ear drum (**a**). Visualization of middle ear by TEES (**b**). The tragal perichondrium graft was placed using an underlay technique (**c**). 1 month postoperative (**d**). 3 months postoperative (**e**). 6 months postoperative (**f**)

All patients underwent general anesthesia with endotracheal intubation. In the TEES group, endoscopic type I tragal cartilage tympanoplasty was performed with a full-thickness graft. The tragal cartilage perichondrium graft was harvested from the tragus, leaving a 2- to 3-mm rim at the dome of the cartilage to prevent deformation of the remaining tragus. The perichondrium was removed from the convex side of the tragal cartilage and an island graft was prepared, with the perichondrium being about 1–2 mm wider than the cartilage. A wedge-shaped incision was made from the center of the cartilage to accommodate the malleolus handle (Fig. 1a–f). Patients in the MES group underwent a post-auricular approach primary type I tympanoplasty that involved harvesting of graft tissue from the temporalis muscle facia. The graft materials were placed using an underlay technique medial to the remnant tympanic membrane. The external auditory canal and middle ear space were packed using gel foam.

### Outcome measures

The main outcome measures were the surgical outcomes, restoration of hearing, and use of medical resources. The surgical outcomes included the successful graft rate and any postoperative complications. The restoration of hearing included pre- and postoperative ABG and average hearing gain (ABG improvement) in decibels. Use of medical resources included the average time spent in surgery in minutes.

External ear canal packing was removed within 3 weeks. Patients received regular endoscope and audiometric evaluation every 3 months for the first postoperative year and then on a yearly basis. A successful graft was defined as full healing of the grafted tympanic membrane, without perforation and retraction, for at least 6 months postoperatively. Audiometric data included preoperative and postoperative air conduction and bone conduction PTAs at each of 4 tested frequencies (500, 1000, 2000, and 4000 Hz). The ABG was calculated from this information. Postoperative outcomes were calculated from the most recent audiogram available.

### Statistical analysis

Data analysis was used SPSS version 23.0. Statistical comparisons were made using the chi-square test, and *t* tests for independent samples. The results were assessed within a 95% reliance and at a significance level of *P* < 0.05.

## Results

### Clinical feature

224 patients (100 female, 124 male) underwent endoscopic Type I tragal cartilage tympanoplasty, while 121 patients (56 female, 65 male) underwent microscopic tympanoplasty with temporalis facia. The mean ± standard deviation (SD) age was 40.87 ± 11.3 years (range 18–67 years) in the endoscopic group and 38.56 ± 11.5 years (range 18–66 years) in the microscopic group; the difference in mean age was not statistically significant (*t* = 1.79; *P* = 0.74) (Table 1).

**Table 1:** Baseline clinical and demographics of patients in two groups

	TEES (*n* = 224)	MES (*n* = 121)
Average age ± SD (years)	40.87 ± 11.3	38.56 ± 11.5
Sex		
Male	124	65
Female	100	56
Lesion side		
Left ear	102	67
Right ear	122	54
Average follow-up period ± SD (months)	12.74 ± 7.56	14.08 ± 6.36
Approach		
Transcanal	224	0
Endaural	0	0
Post-auricular	0	121
Graft material		
Tragal cartilage perichondrium graft	224	0
Temporalis muscle facia	0	121

All patients were followed up for at least 6 months after surgical intervention. The mean ± SD follow-up period was 12.74 ± 7.56 months (range 6–48 months)in the endoscopic group and 14.08 ± 6.36 months (range 6–36 months) in the microscopic group; the difference in mean follow-up was not statistically significant (*t* = − 1.66; *P* = 0.098) (Table 1).

### Comparison of surgical outcomes

The mean ± SD operative time was 49.22 ± 8.24 min (range 30–70 min) in the TEES group and 81.22 ± 14.73 min (range 60–110 min) in the MES group; the difference in mean operative time was statistically significant (*t* = 25.88; *P* < 0.0001; Table 2).

**Table 2:** Outcomes of group TEES and MES

	Group		*P*
	TEES (*n* = 224)	MES (*n* = 121)	
Operative time	49.22 ± 8.24	81.22 ± 14.73	0.000
Tympanic membrane healing			
Postoperative 1 month	219 (97.77%)	118 (97.5%)	0.884
Postoperative 3 months	217 (96.88%)	116 (95.87%)	0.759
Postoperative ≥ 6 months	212 (94.64%)	110 (90.90%)	0.239
Postoperative complication			
Ear pain	22 (9.82%)	101 (83.47%)	0.000
Numbness around the ears	0	24 (19.83%)	0.000
Tinnitus	5 (2.23%)	8 (6.61%)	0.071
Wet ear	0	5 (4.13%)	0.005
Abnormal taste	3 (1.34%)	2 (1.65%)	1.000

The successful graft rates of the TEES group at 1, 3, and 6 (and > 6) months postoperatively were 97.77%, 96.88%, and 94.64%, which were better than the rates in the MES group, which were 97.5%, 95.87%, and 90.90%, respectively. However, the differences were not statistically significant (*P* > 0.05; Table 2).

We analyzed the incidence of postoperative complications (Table 2). Patients in the MES group had a significant higher incidence of wound problems (ear pain, numbness around the ears, and wet ear; *P* < 0.05). However, there were no significant differences in tinnitus and abnormal taste. None of the postoperative patients experienced severe hearing loss or facial paralysis.

### Comparison of hearing outcomes

Preoperatively, the air conduction levels of the operation ear were 37.846 ± 9.41 dB in the TEES group and 36.508 ± 9.83 dB in the MES group. There were no significant differences between the two groups (*P* = 0.216). The ABGs were 19.26 ± 6.43 dB and 18.13 ± 6.01 dB, respectively. There were no statistically significant differences between the two groups (*P* = 0.113).

Postoperatively, the improvements in the postoperative air conduction levels between the 2 groups at 1 month (26.10 ± 7.21 dB and 26.24 ± 7.39 dB, *P* = 0.87), 3 months (26.02 ± 7.29 dB and 26.02 ± 7.33 dB, *P* = 0.999), and 6 (and > 6) months (25.94 ± 7.07 dB and 26.50 ± 7.82 dB; *P* = 0.427) were not significantly different. The improvements in the postoperative ABGs between the 2 groups were not significantly different at 1 month (7.85 ± 4.14 dB and 8.02 ± 4.26 dB; *P* = 0.727), 3 months (7.75 ± 4.11 dB and 7.94 ± 4.26 dB; *P* = 0.681), and 6 (and > 6) months (7.72 ± 4.00 dB and 8.34 ± 4.84 dB, *P* = 0.201). The average hearing gain in the TEES group at 6 (and > 6) months postoperatively was 11.85 ± 5.47 dB, which was better than 10.48 ± 5.18 dB in the MES group (*P* = 0.031). There were no statistically significant differences between the 2 groups at 1 and 3 months postoperatively for average hearing gains (Table 3).

**Table 3:** Pre- and post-operative hearing conditions of two groups

	Group		*P*
	TEES (*n* = 224)	MES (*n* = 121)	
Air condition (dB)			
Preoperative	37.846 ± 9.41	36.508 ± 9.83	0.216
Postoperative 1 month	26.10 ± 7.21	26.24 ± 7.39	0.87
Postoperative 3 months	26.02 ± 7.29	26.02 ± 7.33	0.999
Postoperative ≥ 6 months	25.94 ± 7.07	26.50 ± 7.82	0.427
Bone-condition (dB)			
Preoperative	18.69 ± 1.70	18.38 ± 5.29	0.588
Postoperative 1 month	18.25 ± 4.61	18.22 ± 4.74	0.954
Postoperative 3 months	18.27 ± 4.62	18.24 ± 4.75	0.343
Postoperative ≥ 6 months	18.21 ± 4.57	18.25 ± 4.74	0.948
Air–bone gap (dB)			
Preoperative	19.26 ± 6.43	18.13 ± 6.01	0.113
Postoperative 1 month	7.85 ± 4.14	8.02 ± 4.26	0.727
Postoperative 3 months	7.75 ± 4.11	7.94 ± 4.26	0.681
Postoperative ≥ 6 months	7.72 ± 4.00	8.34 ± 4.84	0.201
Average hearing gain (dB)			
Postoperative 1 month	11.61 ± 5.55	10.40 ± 5.18	0.052
Postoperative 3 months	11.76 ± 5.43	10.71 ± 5.31	0.091
Postoperative ≥ 6 months	11.85 ± 5.47	10.48 ± 5.18	0.031

## Discussion

The goals of tympanoplasty are reconstruction of a health tympanic cavity, closure of the perforation, and optimal restoration of hearing [9]. MES using a post-auricular approach or endaural approach is a traditional and commonly used tympanoplasty technique, with success rates ranging from 83 to 100% [10–16].

However, with the straight-line vision of microscopes, the surgeon cannot visualize the middle ear through the ear canal. As a result, MES approaches frequently necessitate soft tissue retraction, with or without drilling of the bone, to visualize the diseased area satisfactorily [17, 18]. This may prolong the operative time and increase the incidence of postoperative complications [18, 19]. In this study, we observed the mean operative time was significantly longer in the MES group than in the TEES group (*t* = 25.88; *P* < 0.0001). Patients receiving MES had a significantly higher incidence of wound problems (ear pain, numbness around the ears, and wet ear; *P* < 0.05). In a study by Huang et al. [20], the mean operative time was 50.4 min among 50 patients who underwent endoscopic tympanoplasty, compared with 75.5 min for the microscopic approach (*P* < 0.0001). Hsu et al. [21] also reported that the mean duration of surgery and the operative time were shorter in the TEES group than in the MES group, and the postoperative complications (severe sensorineural hearing loss, mastoiditis, and persistent otorrhea) were less common. Choi et al. [16] also reported that patients who underwent TEES experienced significantly less pain at the first day after surgery than patients who underwent MES.

We infer that TEES transforms the external ear canal into an operative area [22, 23], leaving far less soft-tissue damage, no hair loss, and only a tiny wound associated with graft harvesting [24, 25], which may somewhat influence the need for hospitalization [26]. Previous studies [13, 27, 28] used endoscopes instead of microscopes for tympanoplasty and cholesteatoma surgery and noted the following advantages of TEES. Specifically, TEES can provide good illumination, shorten the focal length of the observation object, expand the viewing angle, and explore the “blind area” under the microscope, with minimal interference in the middle tympanum [24, 25].

In our study, the perforation closure rates were achieved in 219 patients (97.77%) in the TEES group and in 118 patients (97.5%) in the MES group at 1 month post surgery. The perforation closure rates were similar in the TEES and MES group with a postoperative follow-up period of at least 6 months (94.64% vs 90.9%, *P* = 0.239). Hsu et al. [21] also reported no significant differences in the graft success rates of in TEES and MES groups (96.2% vs 92.0%, *P* = 0.2826) of patients who underwent tympanoplasty without cholesteatoma. Ohki et al. [29] reported on 47 patients who underwent TEES without ossiculoplasty for chronic otitis media with a 1-year follow-up period. The surgical success rates for tympanic membrane closure were 93.6% for the TEES group and 85.3% for the post-auricular MES group (*P* = 0.244). The surgical success rate for hearing (air–bone gap ≤ 20 dB) was 95.7% in the TEES group and 84.0% in the post-auricular MES group (*P* = 0.077).

These outcomes are consistent with those obtained in our study. The endoscopic approach is less invasive (no skin incisions or canalplasty are required), results in a good graft success rate, and enables better visualization. Compared with the microscopic approach, the endoscopic approach can reduce postoperative bleeding and pain and achieve superior cosmetic results [30]. In addition, minimal damage to healthy structures is likely to minimize postoperative adverse reactions locally and generally.

Both groups in the current study had equal improvements in hearing and ABG. There were no statistically significant differences in the preoperative ABG (dB) (*P* = 0.113) and postoperative ABG (dB) at 1 month (*P* = 0.727), 3 months (*P* = 0.681), and 6 months (*P* = 0.201) between the TEES and MES groups in hearing restoration. We also observed the average postoperative hearing gain (dB) at 1 and 3 months were not significantly different between the 2 groups (Table 3). However, the average postoperative hearing gains at 6 (and > 6) months were better in the TEES group than in the MES group (11.85 ± 5.47 dB vs 10.48 ± 5.18 dB, *P* = 0.031). We assume that the graft source is the reason for this difference because the elasticity of cartilage perichondrium may be superior to that of temporal muscle fascia. However, this result is limited due to the lack of long-term outcomes. Onal et al. [31] evaluated primary tympanoplasty cases with a subtotal perforation to compare the functional results of type I tympanoplasty in patients with bilateral chronic otitis media. The graft success rate for at least 12 months postoperatively was 65.9% for the fascia group and 92.3% for the cartilage group. There was no statistically significant difference in postoperative hearing gain between the two groups (11.63 ± 12.62 dB in the fascia group vs 14.49 ± 10.05 dB in the cartilage group, *P* = 0.268). It was reported that there were no statistically significant differences in the preoperative (21.6 ± 11.2 dB versus 21.4 ± 10.6 dB, *P* = 0.9507) and postoperative (> 6 months) ABG (8.90 ± 10.0 dB versus 8.3 ± 10.0 dB, *P* = 0.7641) between the TEES and MES groups in hearing restoration. Hsu et al. [21] also reported that the average hearing gains following surgery were 10.27 ± 6.4 dB and 12.43 ± 7.46 dB in TEES and MES (*P* = 0.1663).

In our series, we achieved a graft success rate of 94.64% following an endoscopic tympanoplasty at least 6 months, with satisfying improvements in hearing and fewer post-operative complications. To reduce selection bias by adopting TEES and MES in a consecutive series in this study, we selected a clear temporal division (before and since 2013) rather than one based on disease severity. The basic characteristics of the TEES and MES groups were compared, including potential risk factors such as sex and age of the patient, size of the perforation, primary operation, status of the middle ear mucosa, preoperative hearing level, and preoperative air–bone gap. There was no significant difference in any of the parameters. All of the operations in the present study were performed by a single surgeon (W. Zhang) who has been using fascia and cartilage grafts for tympanoplasty for more than 20 years. Thus, we eliminated between surgeon variations in approach as a confounding factor. Although some confounding factors exist, such as different grafts materials and part of the patients in the TEES group were within the learning curve, the results of our study reflect clinical reality to some extent. Therefore, we believed that TEES could achieve surgical outcomes as good as those of MES and reduce postoperative complications and use of medical resources due to a shorter procedure time.

### Limitation

This retrospective study was limited by a number of factors. First, we did not report the other grafting materials in TEES because the data were less and incomplete. Second, MES via endaural approach was not mentioned throughout the study because all of the MES in our hospital were performed by the senior author (W. Zhang) via the post-auricular approach. Finally, this study conducted at a single hospital. A more extensive survey of cases a relatively long-term follow-up results or a multi-hospital study would be beneficial.

## Conclusion

Under favorable conditions, TEES and MES produced comparable hearing outcomes. TEES was a more beneficial approach for hearing gain than MES. With respect to tympanic membrane repair, TEES and MES were comparably effective. However, prospective studies should be conducted in future to confirm these conclusions.

## Abbreviations

PTA: Pure tone average
ABG: Air–bone gap
95% CI: 95% Confidence interval
TEES: Transcanal endoscopic ear surgery
MES: Microscopic ear surgery
COM: Chronic otitis media
